# Enhancement the crystallization and electro-magnetic properties of BaO–Al_2_O_3_–B_2_O_3_ glass ceramics doped with Nd_2_O_3_-rare earth

**DOI:** 10.1038/s41598-023-45131-1

**Published:** 2023-10-25

**Authors:** H. A. Abo-Mosallam, M. A. Azooz, Ebrahim A. Mahdy

**Affiliations:** Glass Research Department, National Research Centre, El Buhouth St., Dokki, 12622 Cairo Egypt

**Keywords:** Chemistry, Materials science

## Abstract

In the present study, a novel glass system containing Neodymium(III) oxide with BaO, Al_2_O_3_, and B_2_O_3_ were created via a popular melt-quenching technique. Nd_2_O_3_ were added, in different concentrations, instead of B_2_O_3_ to study its impact on the crystallization, and electro-magnetic behaviors of the prepared poly-crystalline materials. Thermal characteristics via DTA, XRD and SEM techniques were involved to explore the crystallization and structural properties. The magnetic parameters of the prepared glass–ceramics were studied by VSM measurements. As well the electric properties were also explored. BaB_2_O_4_ and Al(BO_3_) phases were firstly crystallized then Ba_3_Nd(BO_3_)_3_ phase was incompletely precipitated instead of BaB_2_O_4_ phase owing to Nd_2_O_3_ additions. As well, the internal structure was modified from coarse crystals to fine grain microstructure. The crystallization study proved that the addition of neodymium improved the crystallization process of the BaO–Al_2_O_3_–B_2_O_3_ glass system. The VSM and conductivity analysis for the crystalline materials proved that the Nd_2_O_3_/B_2_O_3_ substitutions led to an increase in the electrical and magnetic parameters of the investigated materials. The data obtained from the prepared crystalline specimen showed that these materials are with a distinct and promising ferro-electrical property for use in diverse modern applications.

## Introduction

Nowadays, many world scientists are attracted to developing modern materials to keep pace with the rapid development of our life requirements. A glass and glass–ceramics are one of these gifted materials. Glass–ceramics have caught the interest of academics due to their excellent combination of properties and numerous commercially available products for consumers and specific markets^1^. Glass–ceramics are now defined as nonmetallic, inorganic materials that are created by controlling the crystallization of glass systems using a variety of processing methods^2^. They are composed of at least one functional crystal stage imbedded in a glassy matrix. Among these, borate glasses and their glass–ceramic derivatives have consistently drawn attention and been the topic of numerous studies and publications^3^. They are suggested as potential hosts for radioactive waste^4^, candidates for fast ionization^5^, or materials with electro-optic, 2nd harmonic generation, and nonlinear optical activity. Borate-based glasses have a blend of planar B_3_O_6_ boroxol rings with triangular [BO_3_] units. The concentration of boroxol rings decreases when some alkali or alkaline-earth oxide ions are introduced to the matrix of the borate glass because three-coordinated boron is changed to four-coordinated boron, and the concentration of boroxol rings falls, which increases network connectivity in the structure of the created glasses^6^.

Barium oxide (BaO) is one of the alkaline earth oxides that has a significant impact on the properties when introduced in the borate-based glasses, including phase formation and thermal stability^7^. In barium-borate glasses, BaO can react with B_2_O_3_, leading to the conversion of some [BO_3_] to [BO_4_] in the glass structure. This is a special characteristic of frequently manufactured borate glasses, which exhibit two coordination states in their network structure. These materials possess fascinating, beneficial physical characteristics such as dielectric properties and second harmonic generation^8^.

The crystallization characteristics of barium borate as a binary the glass system in different concentrations have been investigated by several researchers^9–11^. Some studies^8,12–14^ confirmed the effect of TiO_2_ doped in Ba-borate glasses as nucleating agent. Środa et al.^15^ examined the impact of CeO_2_ doping on the structure, thermal stability, and luminescence features of barium borate glasses and glass–ceramics. Glass–ceramics in the barium aluminium borate system were studied by Russel and others^16–18^. Recently, the structural role of Nd_2_O_3_/B_2_O_3_ replacement in the borate glasses and glass ceramics based on the 46B_2_O_3_–27CaO–24.4Na_2_O–2.6P_2_O_5_ (mol%) system has been studied^19^. The amorphous specimens were synthesized through the melt quenching technique. Increasing the Nd_2_O_3_ content at the expense of B_2_O_3_ led to a growth in the glass transition temperature (Tg) and the measured density, which increased the glass hardness. However, the calculated molar volume decreases. The change in the glass batches by an Nd^+3^ large cation rather than a B^+3^ led to a rise in the toughness of the glass network by increasing the bridging bonds in the glass network as a result of decreasing both the molar volume and free spaces of the investigated compositions. The state of crystallization in the high-Nd_2_O_3_-containing glass (4 mol%) is more affected by the glass compositions than by the applied thermal treatment schedule.

The presence of rare-earth oxide (REO) in the glass structure led to increased permittivity, reduced dielectric loss, raised resistivity and a high elastic modulus^20^ and other beneficial electrical and magnetic properties^21–23^. Neodymium oxide (Nd_2_O_3_) is a REO that gives glass unique features such as thermal, mechanical, chemical, optical, and electrical properties^21,22^. Due to these outstanding characteristics, Nd_2_O_3_-containing glasses have already been used in numerous modern applications^23–25^. Neodymium oxide has an astonishingly high melting point of 2300 °C^26^. These characteristics could influence the progress of hermetic materials for sealing purposes at high temperatures or refractory materials with great corrosion resistance. Despite the fact that Nd_2_O_3_ acts as a network modifier, Kohli and Shelby^21^ found that increasing the Nd_2_O_3_ content in aluminosilicate glasses increased the Tg and Td slightly. This behavior is attributed to the main effect of Nd_2_O_3_ as high field strength rather than a glass modifier.

The aim of our research is to examine the impact of the Nd_2_O_3_/B_2_O_3_ replacements on crystallization behavior. The electro-magnetic properties of the new novel poly-crystallized glasses containing Nd_2_O_3_ were prepared via fabricated glasses based on the BaO–B_2_O_3_(Nd_2_O_3_)–Al_2_O_3_ system (mol%), followed by heat treatment according to DTA data. The structural characteristics of these unique glass–ceramics were investigated by the XRD technique and SEM images using five different concentrations. The impact of Nd_2_O_3_/B_2_O_3_ replacements on the density, magnetic parameters, and electric properties was explored to develop new promising electro-magnetic glass–ceramics for future applications.

## Experimental techniques

### Glass and glass–ceramic synthesis

The 20 BaO–(70 − x) B_2_O_3_–xNd_2_O_3_–10 Al_2_O_3_ glass system was carefully synthesized by the melt-quenching method, (where x = 0.5, 1, 2, and 3 mol%). The glass batches of this work are shown in Table 1. High purity grade (˃ 99%) raw materials of BaCO_3_, H_3_BO_3_, Al_2_O_3_, were purchased from Fluka chemei AG, Switzerland, and Nd_2_O_3_ was purchased from BDH chemicals, England were used. The suitable amounts of these compositions were combined and placed in a platinum crucible after being accurately weighted using an electronic balance that had an accuracy of ± 0.0002 g. The mixture was then melted at 1250 °C for 90 min in an electrical furnace. Molds made of hot stainless steel were used to form the desired shapes from the molten glass for experimental measurements. These glass species were then transferred to an electric furnace and annealed at 450 °C for two hours to prevent cracking. Then, the furnace was turned off after the definite amount of time (120 min) of annealing, and the glass specimens were kept inside until the temperature reaches to ambient temperature. Lastly, the solid glass samples were heat treated at specific temperatures per time through double-stage schedules according to DTA data, as presented in Table 2, to prepare glass–ceramic samples.

**Table 1:** The studied glass compositions (mol%).

Composition (mol%)				
Sample ID	BaO	B_2_O_3_	Al_2_O_3_	Nd_2_O_3_
GNd0	20.00	70.0	10	–
GNd0.5	20.00	69.5	10	0.5
GNd1	20.00	69.0	10	1
GNd2	20.00	68.0	10	2
GNd3	20.00	67.0	10	3

**Table 2:** The thermal study and glass–ceramics characterization.

Sample ID	Heat-treatment (°C/h)	∆T = Tc − Tg [Refs.^59,60^]	Crystalline phases	Density (g/cm^3^)	Magnetic properties		
					Ms (emu/g)	Hc (G)	Mr (emu/g)
GNd0	530/2–755/1	225	BaB_2_O_4_, AlBO_3_	2.92	0.104	35	3.07
GNd0.5	548/2–779/1	222	BaB_2_O_4_, AlBO_3_	2.94	–	–	–
GNd1	616/2–786/1	170	BaB_2_O_4_, AlBO_3_, Ba_3_Nd(BO3)_3_	2.99	0.132	65	4.03
GNd2	636/2–789/1	153	Ba_3_Nd(BO_3_)_3_, BaB_2_O_4_, AlBO_3_	3.06	–	–	–
GNd3	652/2–799/1	147	Ba_3_Nd(BO_3_)_3_, BaB_2_O_4_, AlBO_3_	3.11	0.203	91	5.95

### Material characterizations

The powdered glass samples (about 10 mg) will subjected to a differential scanning Calorimetry, (DTA-Netzsch-STA449C, Germany) with a temperature region of 24–800 °C with heating rate of 5 °C per minute using alumina crucible. From the results obtained, it is possible to know the controlled heat-treatment regime at which crystallization occurs, such as the glass transition temperature (Tg) and the crystallization temperature (Tc).

XRD (X-Ray Diffraction) analysis of the heat-treated glasses will performed to determine the crystalline phases precipitated. X-ray diffractometer (XRD) was performed to examine the crystalline phases of each samples using (Philips X-ray diffractometer PW 1730). The Cu-Kα X-ray radiation (λ = 1.5406 A) powered was at 40 kV and 40 mA in the 2θ range of 10°–70° in steps of (2θ) = 0.01.The microstructure morphology of selected crystallized glass-ceramic specimens was studied by Scanning Electron Microscopy (SEM, Quanta 250 FEG-FEI Company, Netherlands). The specimens was detected on fresh fracture surfaces after etched by soaking in 2% HF acid for 45 s.

The Archimedes method will be used to calculate the densities of bulk crystalline specimens with distilled water (density of water, ρw = 1 g/cm^3^) as the immersion liquid. Each sample contained five unique pieces. Using an electrical digital balance with an accuracy of ± 0.02 mg, weight the poly-crystalline specimens in air (Wair) and distilled water (Ww) to determine their respective weights (g). Density was calculated according to the following equation the sample density value, ρ sample, (g/cm^3^):

1 $$\rho {\text{ sample}} = {\text{ Wair }}/ \, \left( {{\text{Wair }} - {\text{ Ww}}} \right). \, \rho {\text{w}}$$

The room-temperature magnetic parameters of the powder glass–ceramic specimens were examined using (VSM, Lake Shore Model 7410, USA). The used magnetic field is 20 kOe. The obtained measurements were used to decide the saturation magnetization (Ms), remanence magnetization (Mr), and coercive field (Hc) of the crystalline samples. The conductivity behavior of the studied crystalline samples was determined by Hitester Impedance Analyzer (HIOKI 3522-50 LCR) with 0.100 V voltage depending on the frequency. In a fully dry state, an alternating electrical current (AC) was used with the 0.1 Hz–2 MHz as frequency range at room temperature. The fine powders of the examined glass–ceramic sample were pressed at room temperature, by using a mass of 5 tons, to prepare the desired disks for the measurements, which then solidified in an electric furnace at 200 °C for 3 h. The surface of the glass–ceramic discs was covered with silver.

### Ethics approval

The study was approved by the Ethics Committee of National Research Centre.

### Consent to participate

A written informed consent was taken from all participants.

## Results and discussion

### Crystallization behavior of the glasses

The crystallization process involves the production of glass–ceramic from glass through a vital step called heat treatment, in which the glass transforms into a polycrystalline material with a fine microstructure and beneficial properties. These materials are called glass ceramics. To do this, the glass must be subjected to the best possible heat treatment conditions for a desired period of time to form a large number of nuclei and then complete the growth of crystals. To determine the ideal condition for heat treating the prepared glass samples, two techniques can be combined or used separately: the conventional method of trial and error, which involves systematic heat treatment of the samples at various conditions to determine the optimal condition, and differential thermal analysis (DTA).

#### Thermal analysis (DTA studies)

**Figure 1:**
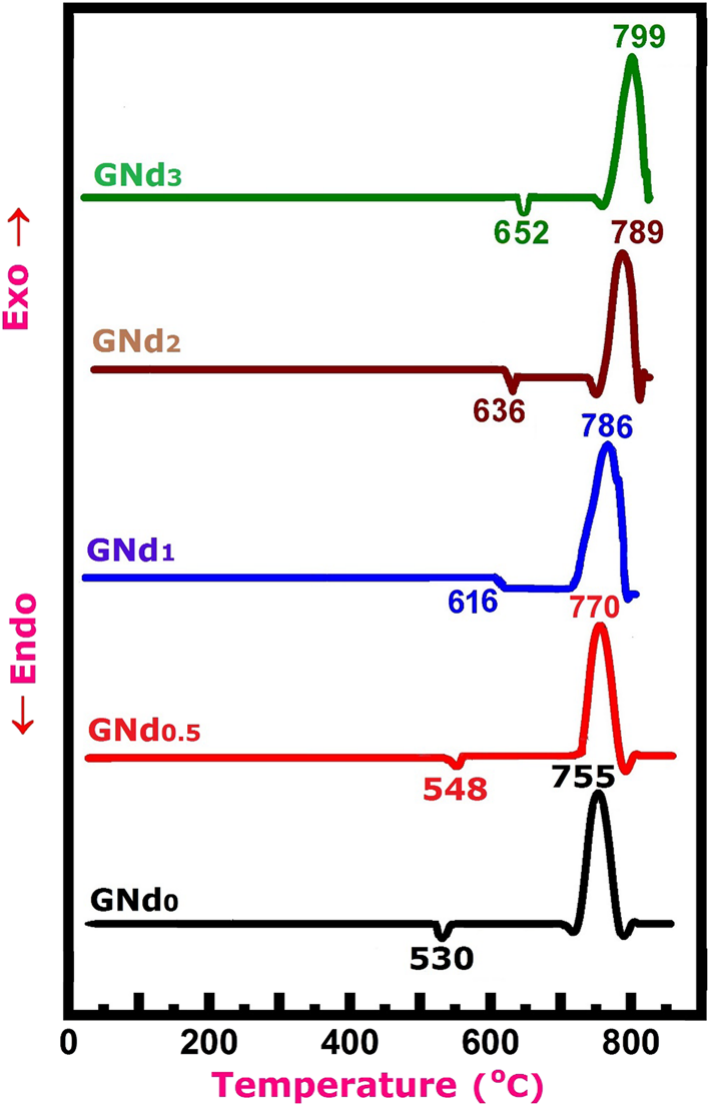
DTA thermographs for the synthesized glass samples.

The DTA technique was used on a fine powder of the studied glass samples to investigate the thermal behavior and determine temperatures of crystallization parameters such as the glass transformation temperature (Tg) and the crystallization temperature (Tc), the values of which are shown in Table 2, and the recorded data is shown graphically in Fig. 1. The measurements were done in temperature ranges up to 900 °C at a rate of 10 °C per minute. The DTA patterns of the synthesized glass samples based on the BaO, Al_2_O_3_, B_2_O_3_, and Nd_2_O_3_ compositions have an interesting feature. The reported data (Table 2, Fig. 1) involved a markedly increased Tg with increasing Nd_2_O_3_ contents in the examined glasses, from 530 °C (for sample GNd0, free of Nd_2_O_3_) to 652 °C (for sample GNd3, with high Nd_2_O_3_ content). As well, the Tc temperatures were slightly increased from 755 to 799 °C for the samples GNd0 and GNd3, respectively.

It is known that Nd_2_O_3_ is a modifier oxide^27^, so this behavior is unexpected. It was anticipated to disorder the glass network, causing a decrease in both the Tg and Tc. The opposite was observed; despite this, given the high field strength of Nd^+3^ ions^27^, this anomaly may be acceptable. Our DTA results are similar to those reported by Kohli and Shelby^21^, which support the suggestion that the effect of Nd_2_O_3_ in the aluminosilicate glasses is in terms of its field strength rather than as a modifier oxide. Additionally, as seen in Table 2, a falling trend of ΔT is found for the Nd_2_O_3_-containing sample, which appears to be related to the rapid crystallization and the distinction in the heat capacities between the base glass, free of Neodymium oxide (GNd0 sample), and the other modified glass samples with different Nd_2_O_3_ content (GNd0.5–GNd3 samples). According to the important principles of the thermal analysis, Gabbott stated that the formed exothermic peaks are related to the thermal impact of crystallization behavior^28^. As a result, the sharp exothermic peaks shown in the DTA curves (Fig. 1) are an indication of the rapid crystallization process taking place over a small temperature interval (Table 2).

#### Crystalline phases formed (XRD study)

**Figure 2:**
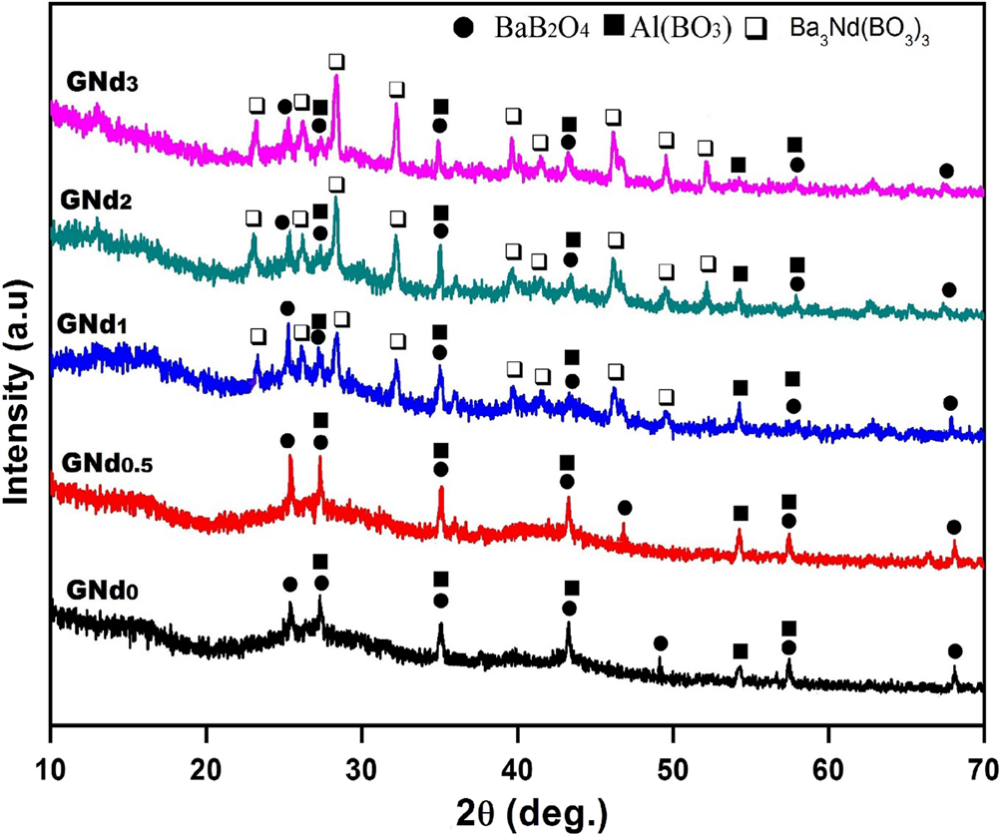
XRD patterns of the studied crystalline samples.

The type and amounts of the crystals precipitated during the crystallization of the glasses in any system depend mainly on the effects of composition, nucleating agents, and the applied heat treatment on both glass formation and. The XRD patterns for the developed crystalline phases as a result of treating glass samples at different temperatures according to the obtained DTA data are presented in Fig. 2, whereas their corresponding phases are recorded in Table 2. It is clear that the diffraction peaks of three different types of crystals formed exactly match their normal diffraction PDF cards. The XRD spectra of the un-doped base glass sample (GNd0) revealed that the control crystallization of the base glass at 530 °C for 2 h and 755 °C for 1 h led to the formation of the BaB_2_O_4_ phase (JCPDS Card No. 44-0584) as a main crystalline phase in addition to the Al(BO_3_) phase (JCPDS Card No. 26-0007) as a minor crystalline phase (Fig. 2, Table 2). The two crystalline phases may be precipitated in the glassy matrix by the use of oxides as in the following Eqs. (2) and (3):

2 $${\text{BaO }} + {\text{ B}}_{{2}} {\text{O}}_{{3}} \to {\text{ BaB}}_{{2}} {\text{O}}_{{4}}$$

3 $${\text{Al}}_{{2}} {\text{O}}_{{3}} + {\text{ B}}_{{2}} {\text{O}}_{{3}} \to {\text{ 2AlBO}}_{{3}}$$

For the glass in the BaO–Al_2_O_3_–B_2_O_3_ system, BaB_2_O_4_ nanocrystals were developed as the major phase during the heat treatment of the glass samples^29^. Additionally, the presence of Al increases the surface tension of the BaB_2_O_4_ melt and extends the glass-forming range^30^. The structure of BaB_2_O_4_ crystals can be seen as a layer-step type of lattice that built up via Ba^2+^ and (B_3_O_6_)^3−^ rings instead^31^.

Doping the studied glass composition (20 BaO, 10 Al_2_O_3_, and 70 B_2_O_3_) with a low content of Nd_2_O_3_ (0.5 mol%) at the expense of B_2_O_3_ (i.e., GNd0.5, heated at 548 °C for 2 h and 770 °C for 1 h), does not add any change in the type or amount of the phases that crystallized in the treated base glass, free of Nd_2_O_3_. This may be attributed to the low content of neodymium oxide (0.5 mol%), which adds to the glass, precipitates in the glass matrix, and is not enough to develop any Nd_2_O_3_-containing phases but the crystallization behavior was improved. These suggestions were confirmed by the obtained XRD results (Fig. 2). Partial increasing of the Nd_2_O_3_ content from 0.5 to 1 mol% (i.e., GNd1) instead of B_2_O_3_ led to the development of the Nd_2_O_3_-containing phase in the form of the Ba_3_Nd(BO_3_)_3_ phase (JCPDS Card No. 51-0425), at the expense of the BaB_2_O_4_ phase, together with the Al(BO_3_) phase. The crystallization of the barium neodymium borate crystalline phase [Ba_3_Nd(BO_3_)_3_] may be formed in a rich media of boric and barium oxide through the reaction described as follows:

4 $${\text{7BaO }} + {\text{ Nd}}_{{2}} {\text{O}}_{{3}} + {\text{ 4B}}_{{2}} {\text{O}}_{{3}} \to {\text{ 2Ba}}_{{3}} {\text{NdB}}_{{3}} {\text{O}}_{{9}} + {\text{ BaB}}_{{2}} {\text{O}}_{{4}}$$

Further increasing the Nd_2_O_3_ content up to 2 and 3 mol% (i.e., GNd2 and GNd3, respectively), the diffraction peak intensity of the Ba_3_Nd(BO_3_)_3_ phase was increased and became the main crystal phase together with the appearance of BaB_2_O_4_ and Al(BO_3_) as minor phases (Fig. 2 and Table 2). The introduction of Nd_2_O_3_ with high content (2 mol% and 3 mol%) in the studied glasses improved the crystallization behavior, but the kinds of crystal phases remained unchanged as shown in XRD patterns (Fig. 2). Furthermore, the growth of Nd-containing crystals adds a further boost to the value of glass ceramics as promising candidates to contain radioactive waste^32^.

#### Microstructural evolution (SEM study)

**Figure 3:**
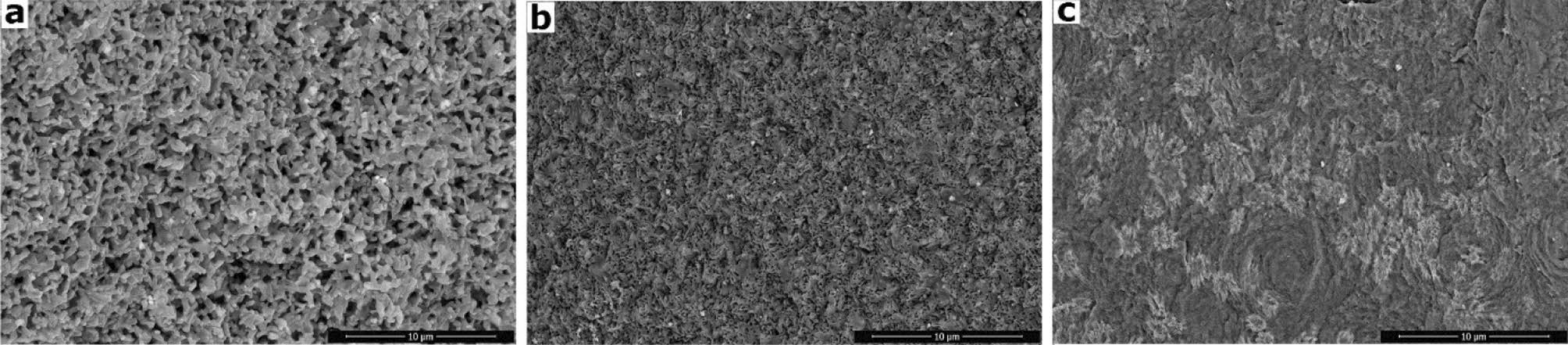
SEM images and micrographs for some crystalline samples.

Microstructure is a powerful factor controlling the properties of glass–ceramic, and it can decrease or increase the features of key phases^33^. The design of an appropriate composition of the glass and the application of a suitable heat treatment control the microstructure developed for the crystalline phases in terms of size and morphology^34^. Figure 3 shows the morphologies of the distribution phases in the selected crystalline samples that were produced by melting and then subjecting them to heat treatment at two different temperatures per specific time as presented in Table 2. The fracture surfaces of the examined samples (GNd0, GNd1, and GNd3) were etched by 2% HF acid for 45 s to reduce the glassy matrix layer. The SEM image of the base crystallized sample, free of Nd_2_O_3_ (GNd0), shows a volume crystallization of dendritic microstructure with micro pores developed inside the formed crystals (Fig. 3a). A denser microstructure of fine prismatic-like crystals was developed, with fine pores formed in between these crystals and distributed in the image of the crystalline GNd1 sample (Fig. 3b). As shown in Fig. 3c, for the sample GNd3, an ultra-fine microstructure was formed with a new generation of fine lath-like growths of Nd-containing crystals that grow as a function of increasing Nd_2_O_3_ content in the glass composition, and this suggestion is evidenced by the XRD patterns for the GNd3 sample (Fig. 2). The observed SEM images show a denser morphology of fine grain microstructure in the three examined samples. However, the average grain size of the crystals decreased with an increase in the Nd_2_O_3_ content (Fig. 3). This observation is in agreement with a previous study by Cheng et al.^35^, who found that the SEM images of the waste doped with Nd_2_O_3_ (0, 10, 19, and 30 wt%) show the formation of very small crystals of micron size and pores formed directly next to these crystals.

### Materials characterization

**Figure 4:**
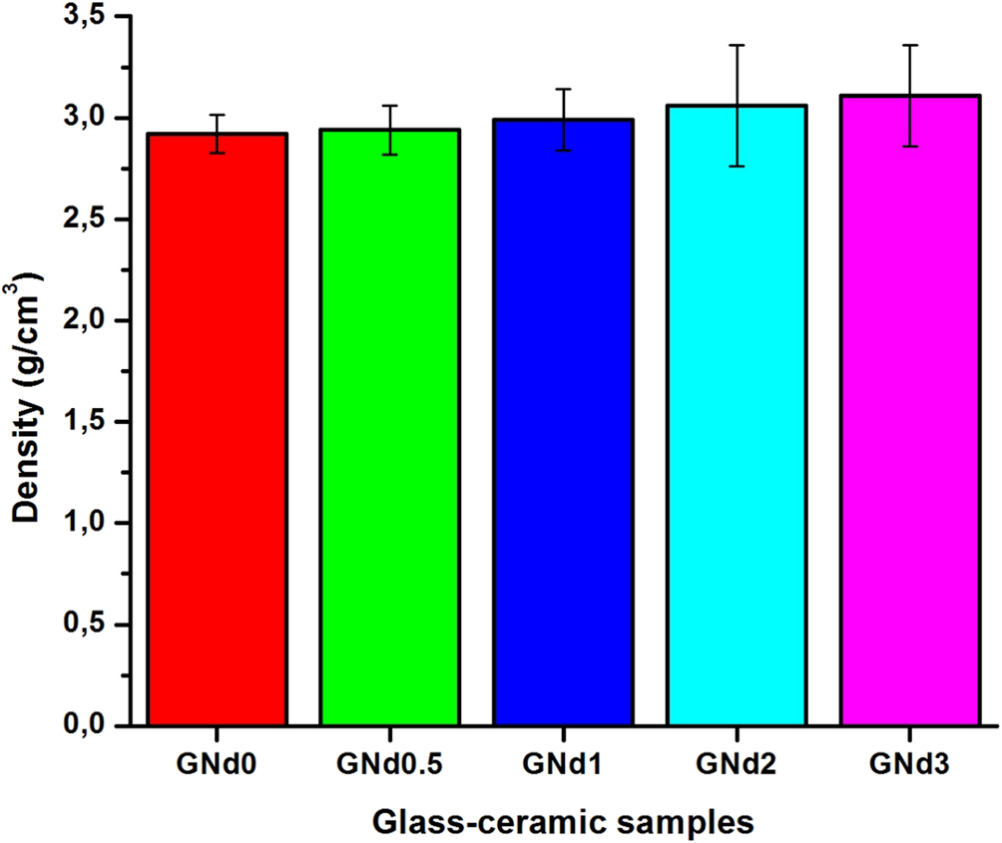
Density of the synthesized glass–ceramic specimens.

Figure 4 shows the density values of the prepared crystalline materials. The data presented that the density values of the crystallized samples were in the variety from 2.92 to 3.11 g/cm^3^. It is clear from the data that with the increase the addition Nd_2_O_3_ instead of B_2_O_3_, the bulk density displays a continuous increase. For the base crystalline specimen (GNd0) the bulk density value is 2.92 g/cm^3^. While, the addition of neodymium oxide up to 3.0 mol% at the expense of boric oxide (GNd3) the bulk density increase to 3.11 g/cm^3^. This may be due to formation of high atomic mass crystalline barium neodymium borate phase Ba_3_Nd(BO_3_)_3_ instead of relatively less dense BaB_2_O_4_ phase. The density of controlled crystallized glasses is affected by a combination of factors, the most significant of which is the density of the crystalline phases formed^36–38^. Another factor may be affecting the density of the synthesized glass–ceramic is formation of fine microstructure with increasing the Nd_2_O_3_/B_2_O_3_ replacement. The density of the crystallized glasses is mainly affected by the granular texture formed^39–41^. Higher bulk density means a condensed microstructure of the glass–ceramic materials^39–41^.

**Figure 5:**
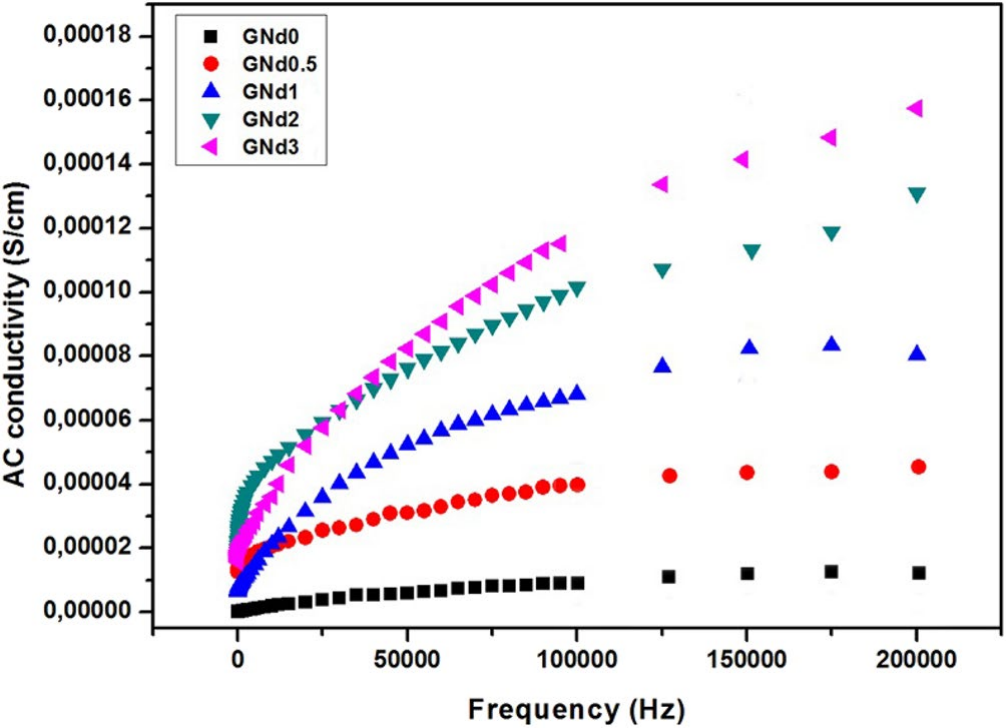
Variation of electrical conductivity with frequency for some crystalline samples.

Recently, interest has increased in the synthesis of new materials that have distinct magnetic and electrical properties^42–44^. This is due to its new and distinct uses in many fields such as electronics, electrolytes, and electrochemical devices^42–44^. Ceramic materials are among the commonly used materials in these fields, especially glass–ceramics, because it has distinct properties that enable it to be used strongly in these fields. The ionic conductivity of the prepared glass–ceramic materials is shown in Fig. 5. The base crystalline sample GNd0 containing BaB_2_O_4_ and Al(BO_3_) as main and secondary crystalline phases respectively showed the lowest conductivity value. It is well known that BaB_2_O_4_ based materials are significant attributed to their amazing nonlinear optical, and piezoelectric properties for technical applications^45,46^. The obtained data indicate that the partial substitution of B_2_O_3_ with Nd_2_O_3_ in the glass specimens led to increase the conductivity values of the corresponding crystalline specimens. The crystalline sample GNd3 with 3 mol% Nd_2_O_3_ showed the highest conductivity value. This may be due to the crystallization of high conducting Ba_3_Nd(BO_3_)_3_ phase on account of the relatively low conducting BaB_2_O_4_ phase. The conductivity of crystallized glasses counts on mainly the type of crystallized phases^47^. The electrical conductivity of barium borate glass doped with Nd_2_O_3_ was studied^48^. They reported that the conductivity of materials increase with increasing neodymium content^48^.

**Figure 6:**
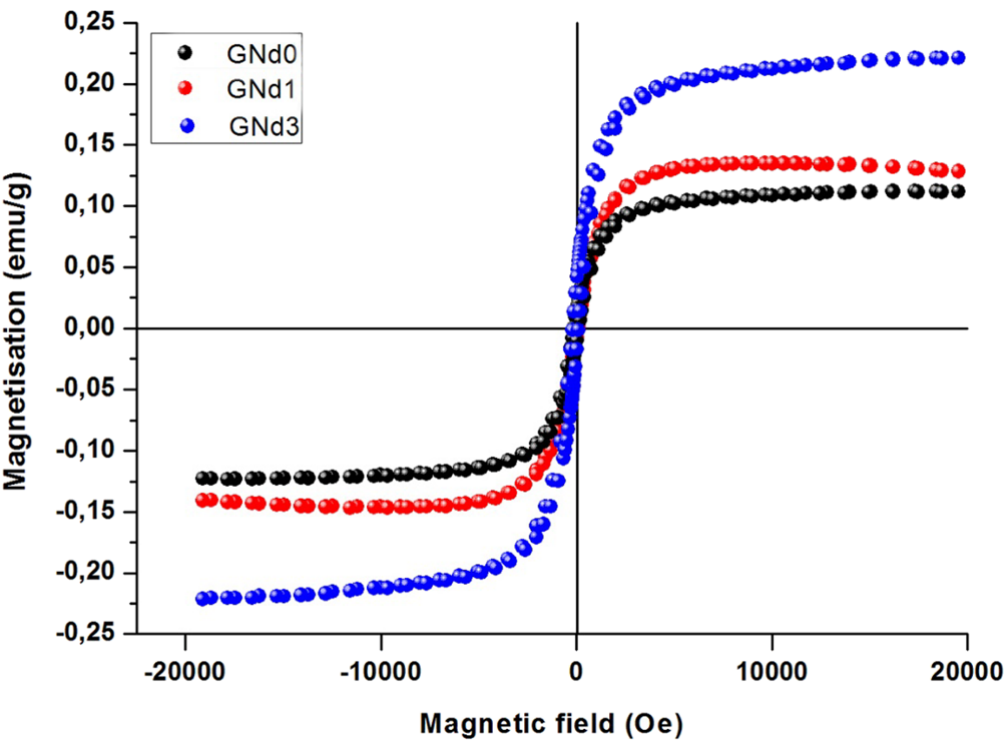
VSM hysteresis loops of the glass–ceramic specimens.

The VSM (Vibrating Sample Magnetometry) hysteresis loops of the synthesized barium aluminum borate glass–ceramics containing different amounts of neodymium oxide is shown in Fig. 6, and Table 2. The magnetic parameters of the crystalline samples were detected at ambient atmosphere under a magnetic field of 20 kOe. In our current study, the focus was on the effect of neodymium content on the magnetic parameters measured of prepared glass–ceramics. The results presented that the measured glass–ceramic samples displayed a soft ferromagnetic attitude. The effects of microstructure as well as crystalline phases designed in the crystallized glasses on the different magnetic parameters, such as saturation magnetization (Ms), remanence magnetization (Mr) and coercivity (Hci), were studied as presented in Tables 2. The (Hci), (Ms), and (Mr) of the crystalline specimens are 35, 87, 91 G, 0.104, 0.132, 0.2039 (emu/g) and 3.07, 4.03 and 5.95 emu/g for GNd0, GNd1, and GNd3 respectively. The estimated magnetic parameters show that the crystalline specimens that Nd_2_O_3_/B_2_O_3_ replacements led to enhance the saturation magnetization (Ms) particularly the sample with high Nd_2_O_3_/B_2_O_3_ replacement ratio i.e., GNd3. This may be due to crystallization of barium neodymium borate Ba_3_Nd(BO_3_)_3_ instead of barium borate phase BaB_2_O_4_ as detected from the XRD patterns (Fig. 2). The change of saturation magnetization in glass–ceramic materials is largely depending on type and amount of crystallized phases^49–53^. Addition of Nd_2_O_3_ led to increase of magnetic properties of barium heksaferit (BaFe_12_O_19_) phase^54^. According the characterization results show that the addition of 0.5%wt.Nd_2_O_3_, the magnetic properties can increase about 40%^55^. On the other side, there is a clear increase in the coercivity and remanence magnetization parameters with addition of Nd_2_O_3_ instead of B_2_O_3_ as shown in Table 2. This may be due to the formation of fine-grained microstructure in the crystalline specimens with increasing Nd_2_O_3_ content, as shown in microstructure images for the crystallized samples in Fig. 3. The formed microstructure and the applied magnetic field are the main and effective factors influencing the values of coercivity and remanence magnetization^56^. When the grain microstructure becomes fine led to increase the coercivity and remanence magnetization parameters^57,58^.

## Conclusion

The formation of novel glasses based on the BaO–B_2_O_3_–Nd_2_O_3_–Al_2_O_3_ system was studied. The principal focus, however, will be on the controlled crystallization of glasses in the studied system. The effect of composition, modifying oxide, and applied heat treatment on both glass formation and crystallization was investigated. The DTA, XRD, SEM, and VSM techniques were used to explore the glass–ceramic materials. As well, density, electric, and magnetic properties are discussed in detail. The obtained Tg and Tc temperatures were increased as a result of the high field strength of Nd_2_O_3_ rather than a glass modifier oxide. The XRD and SEM studies revealed that BaB_2_O_4_, Al(BO_3_), and Ba_3_Nd(BO_3_)_3_ crystalline phases with different morphologies were obtained by modifying the composition with Nd_2_O_3_ dopants. The VSM and conductivity analysis for the crystalline materials proved that the Nd_2_O_3_/B_2_O_3_ substitutions led to an increase in the electrical and magnetic parameters of the investigated materials. The results showed a great potential of the prepared glass ceramics as promising soft ferro-electrical materials that can be used in different modern applications. The obtained data led to further improving and developing the abilities of these Nd-containing glass–ceramic materials with desirable properties for radioactive waste applications in the future.

## Data Availability

Data and materials are all available and prepared, authors will be pleased to provide it if requested during the publication process. The corresponding author (M.A. Azooz) has a permission from other authors to reply if someone wants any data from this study.
